# Earthworm breeding techniques and their roles in microbial regulation and soil carbon sequestration

**DOI:** 10.3389/fmicb.2025.1651602

**Published:** 2025-09-03

**Authors:** Jiahui Wu, Yu Yu, Xueqing Deng, Fuwei Wang, Xianfu Yuan, Hongbao Wu, Jianfei Wang

**Affiliations:** ^1^College of Resource and Environment, Anhui Science and Technology University, Fengyang, China; ^2^Anhui Province Agricultural Waste Fertilizer Utilization and Cultivated Land Quality Improvement Engineering Research Center, Fengyang, China

**Keywords:** earthworm, vermicompost, breeding techniques, microbial regulation, carbon sequestration

## 1 Introduction

Earthworms, as a common soil animal, play an important role in the material cycle and nutrient transformation process of many terrestrial ecosystems (Ngo et al., 2012; Hoeffner et al., 2018). Earthworms can promote the mineralization of nitrogen and phosphorus, and thereby enhance the availability of soil nutrients (Medina-Sauza et al., 2019; Bhadauria and Saxena, 2010). Earthworms enhance soil porosity through their burrowing activities, facilitating soil aeration and drainage while improving soil structure, promoting plant root growth, and enhancing root access to water and nutrients (Ganault et al., 2024). Moreover, enhancing earthworm activity may increase microbial diversity and activity in the soil, accelerating litter decomposition and contributing to the carbon (C) sequestration in soil (Liu et al., 2019).

Vermicompost is the excrement of earthworms, which provides a more stable habitat for microorganisms by regulating pH and cation exchange capacity (Lim et al., 2015). Vermicomposting takes advantage of earthworms during composting to generate an organic material that may be physically, nutritionally, and biochemically improved compared to compost (Akhila and Entoori, 2022). This process converts nutrients in organic matter (such as nitrogen and phosphorus) into forms that are easily absorbed by plants, thereby increasing soil fertility (Lim et al., 2015; Hoeffner et al., 2018; Li et al., 2024).

The role of earthworms in shaping soil microbial communities and regulating C cycling has emerged as a central focus in soil ecological research (Ahmed and Al-Mutairi, 2022; Thomas et al., 2020). However, in recent years, the population of earthworms in the soil has gradually decreased due to intensive agricultural activities. So, how to adopt scientific and reasonable earthworm breeding techniques has become a key issue (Pelosi et al., 2013). Thus, this article aims to provide a theoretical basis and technical guidance for earthworm breeding techniques, and deeply analyze the roles of earthworms in microbial regulation and soil C sequestration.

## 2 Earthworm breeding techniques

The selection of a suitable earthworm species is important for breeding (Butt, 2008). Currently, there are over 6,000 species of earthworms in the world (Singh et al., 2020). Ecologically, earthworms can be classified into three categories: epigeic, endogeic, and anecic species, based on the living behavior (Wasunan et al., 2023). Briefly, epigeic earthworms survive on the surface of soil and consume surface organic matter, endogeic earthworms burrow horizontal galleries to feed on soil organic matter, and anecic earthworms burrow vertical galleries to feed on a mixture of surface and deep soil organic matter (Hoeffner et al., 2018). Among them, epigeic earthworms are the most suitable for converting organic matter, while anecic earthworms are more suitable for use as high-protein feed for livestock and poultry (Qiu, 1999; Rong et al., 2020). The *Eisenia Fetida* belongs to the epigeic earthworm, which has a strong ability to decompose organic wastes such as livestock manure and urban sludge, and is suitable for breeding with manure (Yadav and Garg, 2011). In addition, the *Ohira* II earthworms can withstand low temperatures and can still move at −10 °C, making them suitable for high-density breeding in cold regions like Northeast China (Liu et al., 2021). The *Pheretima Guillelmi* has a relatively large body size, prefers organic wastes and fertile soil, and is suitable for breeding in vegetable fields and areas with sufficient feed (Lin et al., 2024).

**Figure 1:**
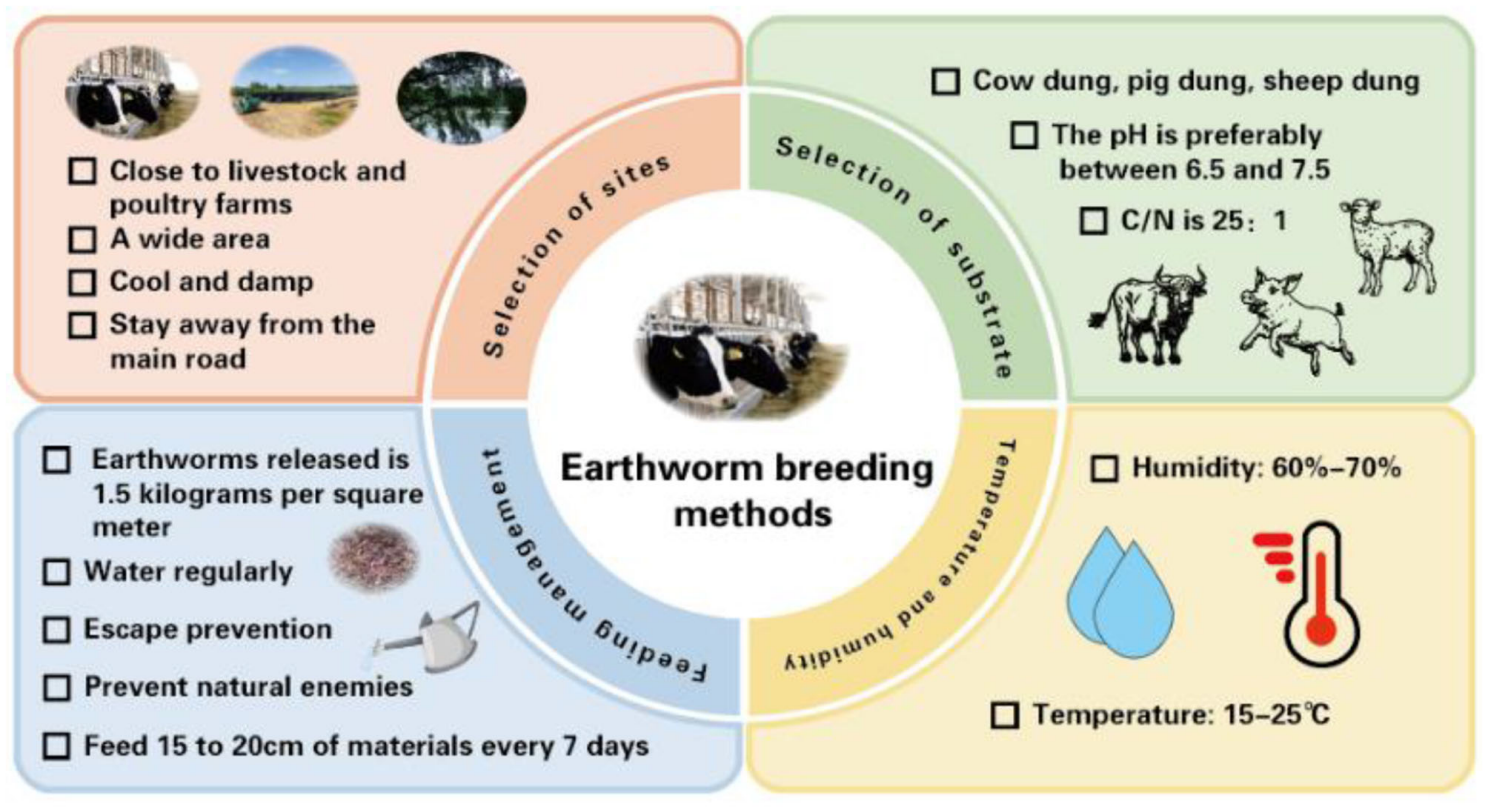
Earthworm breeding techniques.

Apart from species selection, earthworm breeding techniques involve inoculation density, breeding substrate, breeding environment, etc. (Figure 1). Different densities of earthworms are closely related to their growth and development. The appropriate density of earthworms is 1.5 kg/m^2^ (Wang et al., 2023; Xiao et al., 2023). Appropriate temperature, humidity, and sufficient food can reduce the occurrence of earthworm escape (Presley et al., 1996; Chen and Zhong, 2022). To prevent natural enemies, 5% lime can be scattered around the farm to form a defense line (Yi, 2018).

Livestock and poultry manure are commonly used as the substrate for earthworm breeding (Bakayoko et al., 2009). The breeding site should be close to livestock and poultry farms or in an open area that is convenient for storing raw materials (Meng, 2018; Xiao et al., 2025), which facilitates breeding substrate acquisition. Cow manure is more beneficial for the growth of earthworms, the production of vermicompost, and the optimal maturation of the substrate compared to pig manure and sheep manure (Vodounnou et al., 2016; Wang et al., 2022). The C/N ratio and pH value in the substrate are important factors affecting the growth and reproduction of earthworms. The optimal C/N ratio is 25:1, and the pH value typically ranges from 6.5 to 7.5 (Sonia et al., 2016; Behera, 2018). During the breeding process, feed is usually added once a week, with a cover depth of 15–20 cm (Liu, 2023).

Temperature and humidity are also key factors for the growth and reproduction of earthworms. Earthworms prefer to grow in cool and humid environments (Grant, 1955; Ma et al., 2019). To provide the most suitable environment for the growth and reproduction of earthworms, the moisture content of the substrate should be monitored daily (Perreault and Whalen, 2006; Han et al., 2024). The temperature range most suitable for earthworm growth is typically 15–25 °C, and the humidity is approximately 60–70% (Baker and Whitby, 2003; Li et al., 2020; Yin et al., 2022).

## 3 The roles of earthworms in microbial regulation and soil C sequestration

Earthworms constitute the dominant biomass of invertebrates in soil and are known as the “engineers of the soil ecosystem” (Blouin et al., 2013). Earthworm activities create dynamic microenvironments that significantly alter the composition and function of soil microorganisms, ultimately affecting C sequestration processes (Dempsey et al., 2013; Medina-Sauza et al., 2019). A previous study showed that inoculating different ecological types of earthworms increased the diversity of soil fungal communities, thereby enhancing soil quality (Zhang et al., 2025). Earthworms have a promoting effect on enzyme activity and increase the diversity and uniformity of bacterial communities, thereby effectively improving soil quality (Xu et al., 2021). The vermicompost is rich in abundant microorganisms, which can be applied to the soil as a biological fertilizer, thereby increasing soil enzyme activity and improving soil fertility (Gao et al., 2015; Zhao et al., 2023). Many studies have shown that the addition of vermicompost can increase the richness of soil microorganisms (Lim et al., 2015; Singh et al., 2020; Tan et al., 2021).

Earthworms play a pivotal role in regulating soil microbial communities through multiple pathways. First, earthworms increase soil porosity by creating channels through their digging, allowing oxygen to penetrate more easily and improving the water infiltration and retention capacity (Medina-Sauza et al., 2019). The balance between ventilation and water retention provides a more suitable environment for microorganisms, thereby regulating the composition and function of microbial communities (Lim et al., 2015). Second, earthworms promote the decomposition of organic matter and nutrient cycling, enhance soil fertility, and enable plants to obtain more nutrients during the process of burrowing and feeding (Thejesh, 2020; Ahmed and Al-Mutairi, 2022). These alterations in soil fertility and plant growth may regulate the microbial community. Third, through the digestive processes of earthworms, the excreted vermicompost can elevate the content of nitrogen, phosphorus, and potassium and increase the soil water retention capacity, which may affect microbial communities (Lim et al., 2015; Turab et al., 2023).

Earthworms play a fundamental role in soil C sequestration through their unique biological activities that transform organic matter into more stable forms (Don et al., 2008; Zhang et al., 2013; Meng, 2022). Their contribution to C storage operates through multiple pathways, primarily involving the physical breakdown of plant residues, microbial stimulation, and the formation of organo-mineral complexes (Zhang et al., 2013; Angst et al., 2017; Thomas et al., 2020). As earthworms consume and digest organic materials, they accelerate decomposition while simultaneously creating microenvironments that favor C stabilization (Lubbers et al., 2017; Angst et al., 2017). The gut passage of organic matter introduces enzymes and gut-associated microbes that chemically modify C compounds, often leading to the formation of humic substances with greater resistance to microbial degradation (Angst et al., 2017; Thomas et al., 2020). The mucus secreted by the earthworm intestines combines with soil particles and organic matter to form more stable aggregates (Lavelle et al., 1997; Angst et al., 2017). Studies have shown that the adhesive properties of polysaccharides and fungal hyphae largely determine the formation of large aggregates, where the fungal hyphae can prevent certain compounds in the soil aggregates from being utilized by microorganisms, thereby making the C more stable (Samuel et al., 2008; Thomas et al., 2020).

**Figure 2:**
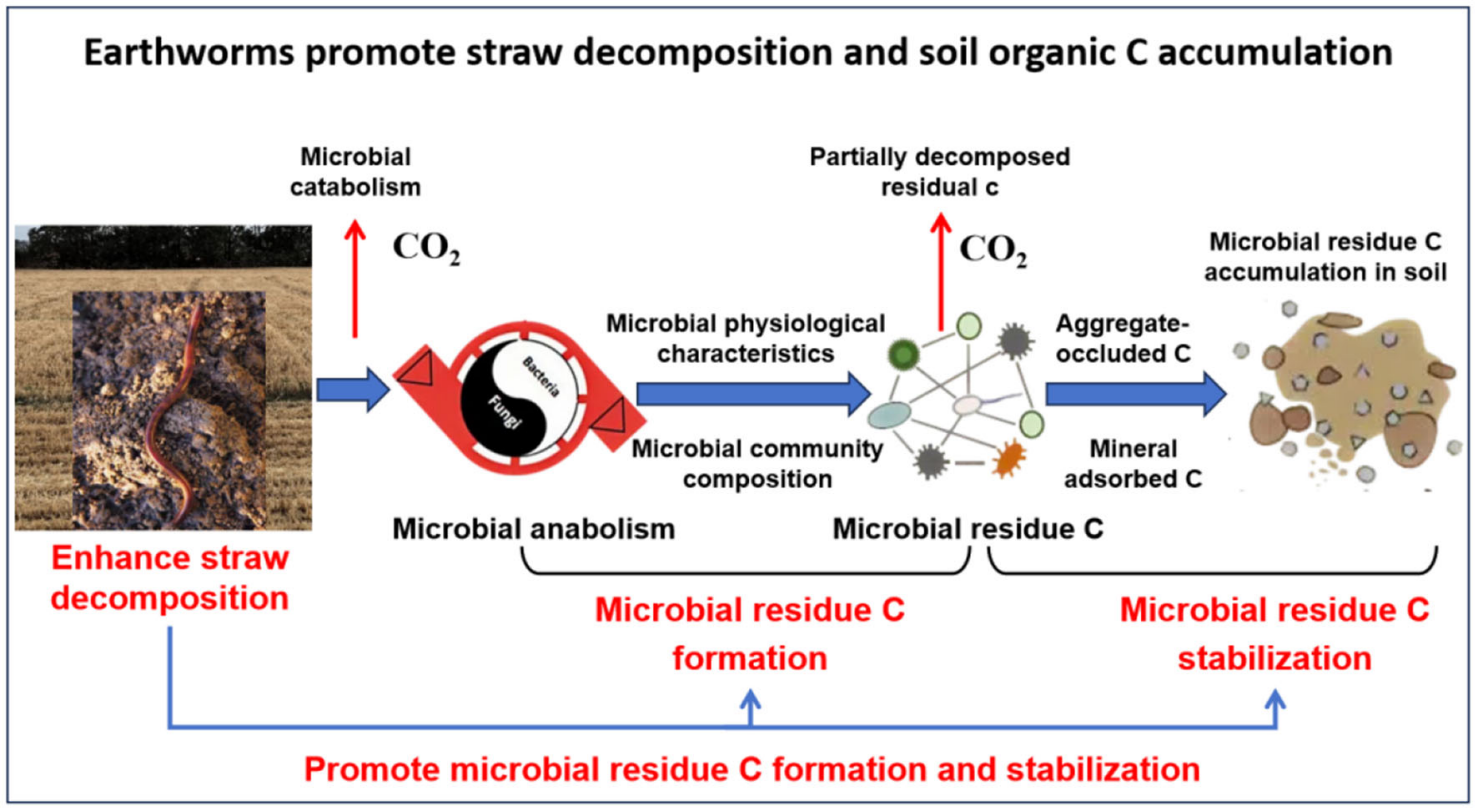
Conceptual framework of earthworms on straw decomposition and soil organic C accumulation.

Based on the roles of earthworms in microbial regulation and soil C sequestration, we propose a conceptual framework of earthworms on straw decomposition and soil organic C accumulation in farmland soils (Figure 2). Earthworms stimulate microbial activity by secreting mucus, break down straw to increase the contact area, and mix organic matter with the soil, thereby accelerating straw decomposition and promoting the combination of microbial residue C with soil minerals, ultimately significantly increasing soil C accumulation (Figure 2).

## 4 Conclusions and prospects

Earthworms and their products play an important role in the improvement of soil fertility in terms of the physical and chemical properties of the soil. We summarized the optimal earthworm breeding techniques involve species selection, inoculation density, breeding substrate, breeding environment, etc. These scientific earthworm breeding techniques provide the foundation for the production of earthworms and vermicompost efficiently in the future. In addition, we synthesized the roles of earthworms in microbial regulation and soil C sequestration to provide a theoretical basis for the application of earthworms in agriculture. Although earthworms can promote the activity of soil microbes and organic matter decomposition, however, whether this decomposed C can persist stably in the soil for a long time remains largely uncertain. Therefore, future research can focus on the potential and mechanisms of earthworms on soil C stability in the long term.
